# Feasibility and preliminary outcomes of compassion-focused acceptance and commitment therapy delivered via telehealth in a community behavioral health clinic

**DOI:** 10.3389/fpsyg.2025.1509396

**Published:** 2025-04-11

**Authors:** Keryn Kleiman, Donald R. Marks, Jennifer Block-Lerner, Dennis Tirch, Victoria Brady, Benjamin Foote, Laura Silberstein-Tirch

**Affiliations:** ^1^Department of Advanced Studies in Psychology, Kean University, Union, NJ, United States; ^2^The Center for Compassion Focused Therapy, New York, NY, United States

**Keywords:** compassion, acceptance, mindfulness, shame, self-criticism, anxiety, depression

## Abstract

**Introduction:**

Given the significant roles self-criticism and shame can play in the development and maintenance of psychological disorders, several compassion-based treatments, such as compassion-focused therapy (CFT), have been developed in recent years to address shame-based difficulties across a range of psychopathological conditions. CFT aligns with major tenets of acceptance and commitment therapy (ACT), which has been shown to be effective in treating various clinical disorders. Compassion focused acceptance and commitment therapy (CFACT) merges elements of CFT and ACT.

**Method:**

This study examined the feasibility, acceptability, and preliminary outcomes of a manualized CFACT protocol for transdiagnostic presentations in a community behavioral health clinic through a non-concurrent multiple baseline single-case experimental design. Participants received the therapy over 16–19 weekly sessions. Symptom severity, self-compassion, guilt, shame, attributional styles (detachment and externalization of blame), psychological flexibility, functioning in valued life contexts, and quality of life were assessed across baseline and treatment phases. Ratings of perceived utility and other aspects of interest/receptivity were also collected.

**Results:**

Results indicate strong acceptability and receptivity for CFACT across both participants and clinicians. Training clinicians in CFACT and implementing the treatment over telehealth in a training clinic setting was feasible. Most participants exhibited reliable decreases in symptom distress and psychological inflexibility, and reliable increases in self-compassion. Detachment level increased for most participants. Trajectories of guilt-proneness, shame-proneness, externalization of blame, and quality of life either varied across participants or remained unchanged. Supplemental cross-lagged correlation analyses did not demonstrate predictive associations between variables.

**Discussion:**

While quantitative outcome results were mixed, preliminary evidence suggests CFACT contributes to reduced symptom distress and increased psychological flexibility, self-compassion, and detached attributional style. Limitations and future directions are discussed.

## 1 Introduction

Compassion-focused therapy (CFT) was developed by Paul Gilbert (2009a) as an integrated treatment to address shame-based difficulties transdiagnostically. Gilbert observed that many individuals exhibiting high levels of shame and self-criticism who had engaged in traditional cognitive therapy were adept in various cognitive and behavioral strategies, such as cognitive restructuring, but their improvement in terms of emotional responses, distress, and impairment was limited. Gilbert posited that their limited progress was largely due to their difficulty in generating feelings of safeness, contentment, and inner warmth (Rector et al., 2000). CFT, therefore, was developed to promote these abilities through the cultivation of compassion for oneself and for others (Gilbert, 2009b).

Compassion-focused therapy’s theoretical foundations draw from neuroaffective science and evolutionary, social, developmental/attachment, and Buddhist psychologies (Gilbert, 2009b). Three evolved affect regulation systems are thought to underpin emotions and social processes: the threat detection and protection system; the drive system; and the contentment, soothing, and social safeness system. Hypersensitive threat detection/protection and drive systems are common in those who experience high levels of self-criticism, guilt, and shame, which are correlated with histories of abuse, neglect, and lack of affection (Andrews, 1998; Gilbert, 2009b). Relatedly, these individuals tend to have difficulty accessing the contentment and soothing system, often due to its under-stimulation early in life (Gilbert, 2009b). Therefore, a primary objective in CFT is to balance the systems by fostering the contentment and soothing system through the cultivation of compassion, which serves to regulate the threat detection/protection and drive systems (Gilbert, 2009b). In CFT, compassion is defined as consisting of two dimensions (Gilbert and Choden, 2013). The first, the psychology of engagement, refers to a sensitivity to and awareness of suffering. The second, the psychology of alleviation, refers to a commitment to engaging with suffering to alleviate or prevent it. Further, compassion is conceptualized as being composed of certain attributes and skills, which are cultivated through the therapeutic relationship and compassionate mind training, which refers to a set of strategies and exercises meant to aid in the development of compassion for the self and others (Gilbert and Irons, 2005; Gilbert, 2009b; Tirch et al., 2014). A number of studies have provided empirical evidence for CFT’s effectiveness in treating a range of conditions, including mood and anxiety disorders (Leaviss and Uttley, 2015; Wilson et al., 2018), substance use disorders (Held et al., 2018), and OCD (Petrocchi et al., 2021).

Acceptance and commitment therapy (ACT) is a third-wave cognitive behavioral therapy that promotes acceptance and mindfulness of inner experiences and commitment to actions that align with personal values and meaningful life directions (Hayes et al., 1999). ACT does not aim to eliminate or reduce negatively-evaluated experiences, but to change the individual’s relationship to these experiences, thereby fostering psychological flexibility and enabling the individual to act according to personal values even when difficult inner experience arises (Walser et al., 2012). Empirical evidence for ACT’s transdiagnostic efficacy for conditions including depression, anxiety (Twohig and Levin, 2017), and PTSD (Pohar and Argáez, 2017), has grown exponentially over recent years, with over 1,000 randomized controlled trials published to date (Hayes, 2023).

Compassion-focused therapy and other compassion-based treatments align with major tenets of ACT (Tirch et al., 2014). CFT shares several key elements with ACT, such as mindfulness, non-judgment, adaptability, openness, and valued action. A main distinction between the two is that CFT places the cultivation of compassion at the center of its model, while ACT does not. Nevertheless, ACT developer Steve Hayes (2008) as cited in Tirch et al. (2014) has suggested that compassion may be the one value inherent in the model of psychological flexibility. Moreover, processes underlying compassion and psychological flexibility are interrelated. For example, compassion necessitates being sensitive to and turning toward suffering, including one’s own painful inner experiences (Gilbert, 2009a; Neff, 2003). This process involves awareness, acceptance, willingness, and perspective-taking. Further, compassion requires not being dominated by attachment to these experiences (or judgments of ourselves or of these experiences) and committing to taking action to alleviate this suffering. Essentially, elements of compassion are inherent in psychological flexibility and vice versa, with the cultivation of one domain potentially promoting growth in the other. Researchers have found that, when compared to a wait-list control condition, ACT resulted in greater increases in self-compassion (Yadavaia et al., 2014). Further, psychological flexibility was found to be a significant mediator for change in self-compassion. Yet, while compassion may be implicit in the ACT model, it has not historically been targeted explicitly.

Some researchers and clinicians have begun to combine elements of compassion-based therapies with ACT to address psychological difficulties in specific populations. Skinta et al. (2015) incorporated ACT with elements of CFT in an 8-session group intervention designed to address stigma associated with HIV. Findings from this pilot study – the first to integrate CFT and ACT – included reduced self-stigma in men with HIV who completed the group. A compassion-focused ACT protocol for women with eating disorders in an outpatient setting found that clinically relevant behaviors appeared to improve across participants (Hill et al., 2020). Azizi et al. (2021) found that providing an intervention combining ACT and CFT principles to mothers of children with hearing impairments resulted in significant improvements in parent–child relationships and reductions in the children’s behavioral problems. Koryani et al. (2022) found that individuals with multiple sclerosis who received a compassion-focused ACT intervention exhibited significantly reduced psychological distress and increased psychological flexibility when compared to controls. In a preliminary examination of a group intervention for sexual minority individuals combining elements of ACT and CFT, as well as other mindfulness-based interventions (Seabra et al., 2024), participants demonstrated reduced stress, social anxiety, self-criticism, and fear of compassion. The results of these preliminary studies suggest that combining compassion-based treatments and ACT is a promising line of investigation. It should be noted that several other group intervention studies have addressed compassion and self-compassion processes in the context of ACT (e.g., Carvalho et al., 2022; Ferreira et al., 2024; Peymannia et al., 2018; Pinto-Gouveia et al., 2017).

With an understanding of the theoretical overlap and shared values of CFT and ACT, Tirch et al. (2014) have developed a transdiagnostic compassion-focused acceptance and commitment therapy (CFACT), merging elements of the two therapies. They propose that targeted, deliberate cultivation of compassion can enhance and expand the scope of therapeutic psychological processes fostered in ACT. They explain that in CFT, the cultivation of compassion is viewed as a process that contributes to more adeptly and flexibly responding to emotions, as well as increasing well-being. This is compatible with ACT’s fundamental theories and aims (Hayes et al., 1999). Moreover, principles in ACT and its underlying theory of functional contextualism (Biglan and Hayes, 2015) can enhance processes involved in the cultivation of compassion. ACT’s emphasis on understanding and predicting behavior, including verbal behavior, can broaden our understanding of processes involved in compassion and strengthen the effectiveness of compassion-focused treatments.

Tirch et al. (2014) propose *compassionate flexibility* as the central process-based model of CFACT. The model encompasses the two dimensions of compassion (psychologies of engagement and alleviation), as well as processes contributing to psychological flexibility. Compassionate flexibility is composed of six processes: sensitivity, sympathy/empathy, non-judgment, distress tolerance, care for well-being, and committed action to alleviate suffering. The developers elaborate on how these processes reflect both the attributes of compassion, as well as elements of the ACT hexaflex. Sensitivity in CFT means turning toward suffering, which relates to present-moment and mindful awareness of whatever experiences arise, including painful ones. Sympathy and empathy both require flexible perspective-taking, which relates to self-as-context. Non-judgment, which promotes the willingness to remain in the presence of suffering, relates to acceptance of present-moment experience. Distress tolerance may be promoted by defusion and acceptance in that both processes increase the willingness to stay in contact with painful experiences. Care for well-being represents a guiding, central value. While values in ACT are freely chosen and idiosyncratic (Hayes et al., 1999), the specific motivation to care for the self and others is central to CFT and compassionate flexibility. These processes are all in the service of committed action for the purpose of the alleviation of human suffering.

Despite the potential benefits offered by the combination of compassion-focused and acceptance-based approaches in Tirch et al. (2014) transdiagnostic CFACT model, the treatment had not been evaluated prior to this study. Further, empirical examination of compassion-focused acceptance and mindfulness-based treatments is limited, and prior to this study, researchers had only investigated the impacts of these interventions in specific populations (e.g., individuals with eating disorders, chronic illness). Moreover, none had examined interventions delivered through telehealth. We, therefore, aimed in this study to examine the feasibility, acceptability, and preliminary outcomes of telehealth-delivered CFACT for individuals with transdiagnostic presentations through a non-current multiple baseline study in a community mental health training clinic setting. Client trajectories across the following variables collected over the course of the study (baseline and treatment phases) were analyzed: symptom severity, self-compassion, guilt, shame, attributional styles (detachment and externalization of blame), psychological flexibility, functioning in valued life contexts, and quality of life. Ratings of perceived utility and other aspects of interest/receptivity were provided by both participants and clinicians. Qualitative data were collected to contextualize quantitative results and provide a deeper understanding of participants’ and clinicians’ experiences at an individual level, which may help inform future iterations of CFACT.

We expected that both clinicians and participants would find the treatment acceptable and appropriate and that clinicians would find the implementation of the intervention feasible. We predicted that clinicians’ adherence to the treatment manual would range from moderate to high. Additionally, the majority of participants would complete the treatment, further supporting evidence of the feasibility and acceptability of this CFACT protocol. Relative to baseline, we predicted that participants would demonstrate increased psychological flexibility and compassion, and decreased symptom distress following the implementation of CFACT. Quality of life was expected to increase. We predicted levels of shame-proneness and guilt-proneness would decrease, detachment would increase, and externalization of blame would decrease. Those exhibiting higher levels of shame and self-criticism at baseline were expected to find CFACT to be especially relevant and useful, aligning with extant literature (Leaviss and Uttley, 2015). We predicted this would be reflected by measures directly assessing interest and perceived utility, as well as by patterns in process and outcome measures.

## 2 Method

### 2.1 Participants, recruitment, and setting

The study took place at an outpatient mental health clinic connected to a doctoral psychology program in a semi-urban area in the northeastern United States. The clinic offers no-cost therapy services to the community. We recruited treatment-seeking adults from the surrounding community. Potential participants seeking services at the clinic, who presented mainly with anxiety, depression, and trauma-related difficulties were screened for appropriateness for the study through a brief phone interview. Exclusionary criteria included: severe mental or physical health issues (psychotic symptoms, substance abuse, eating disorders, or active suicidality), requiring services in a language other than English, and starting or modifying a dose of a psychotropic medication while in the study. Sufficient baseline stability across at least two of three weekly-administered measures (DASS-21, AAQ-II, and SCS-SF) was required for inclusion in the study to enable data analysis. Participants were included if up to one of three baselines demonstrated insufficient stability, but this was taken into account during analysis. Participants who did not meet the baseline stability criterion were still offered treatment by the clinic but were not included in the study.

No prospective participants met criteria for the excluded diagnoses (i.e., severe mental illness, substance use disorders, and eating disorders). Fourteen adults enrolled in the study between March 2022 and April 2023. Of those, three dropped out of treatment prior to completion. Two completed cases were not included in the study analyses due to Internet connectivity problems during telehealth sessions or technical difficulties with video recording sessions for adherence coding. Two cases were excluded due to insufficient baseline stability and two cases did not meet treatment adherence criteria. As a result, a total of *N* = 5 cases were analyzed for the study. Participant characteristics can be found in Table 1.

**Table 1:** Participant characteristics.

**Participant**	**P1**	**P2**	**P3**	**P4**	**P5**
**Gender**	Female	Female	Female	Female	Male
**Age Bracket**	25-34	45-54	55-64	25-34	25-34
**Race/Ethnicity**	White	White/Jewish	White	Asian	White
**Religion**	Atheist/Agnostic	Jewish	‘None’	Atheist/Agnostic	Not reported
**Sexuality**	Declined to report	Heterosexual	Heterosexual	Bisexual	Heterosexual
**Relationship & Parental Status**	Engaged, no children	Married, with children	Married, with children	Married, no children	In long-term relationship
**Occupational Field**	Social Services	Education	Education	Community Development	Retail
**Reported History of Potentially Traumatic Event**	Yes	Yes	No	Yes	Yes
**DSM-V Diagnosis**	Parent-Child Relational Problem (between P1 and her parent)	Adjustment disorder with mixed anxiety and depressed mood, acute	Generalized Anxiety Disorder; ADHD (by history)	Unspecified Mood Disorder with mixed features	Unspecified anxiety disorder; major depressive disorder, in partial remission; ADHD (by history)

### 2.2 CFACT protocol

The CFACT protocol was developed by the authors and was adapted from an unpublished group therapy manual by Tirch et al. (2019). The protocol consists of 16–19 60-min sessions. Protocol content was drawn from *The ACT Practitioner’s Guide to the Science of Compassion* (Tirch et al., 2014), as well as several CFT (e.g., *The Compassionate Mind Guide to Overcoming Anxiety;* Tirch, 2012) and ACT (e.g., *Experiencing ACT from the Inside Out;* Tirch et al., 2019) resources. The sessions were designed to promote each of the processes within the compassionate flexibility model. Session structure was organized as follows: Sessions 1–3: Introduction to Mindfulness (Present-Moment-Focused Sensitivity), Acceptance, and Compassion; Sessions 4–5: Care for Well-Being; Session 6: Building Willingness and Distress Tolerance; Session 7: Observer Self and Flexible Perspective-Taking; Sessions 8 and 10: Non-Judgment/Defusion from Judgment; Sessions 9, 11 and 12: Empathy and Flexible Perspective Taking; Session 13: Sympathy/Emotional Perspective-Taking; Sessions 14–15: Committed Compassionate Action; and Session 16: Sustaining Compassionate Commitment. Since all the processes within the model are interrelated, elements of multiple processes may be present to an extent in any single session. While the protocol consisted of 16 modules which could be delivered over 16 sessions, clinicians were provided the flexibility to extend treatment by a maximum of three sessions in the case that they were unable to complete all required protocol material in the prescribed sessions. Psychoeducation from a CFT perspective [i.e., the 3-circle model of affect regulation, and the message of the “wisdom of no blame” (Tirch et al., 2014)] was provided in initial sessions. Each session incorporated a mindfulness and/or compassionate mind training practice, other experiential exercises from CFT (e.g., the “multiple selves” and the three-chair exercises and compassion-focused visualizations) and ACT [e.g., creative hopelessness, values clarification, and “externalizing (defusing) and thanking the mind”], as well as collaborative discussions. From the third session onward, clients were instructed to identify a values-consistent action at the end of each session to carry out prior to the next session. The clinician used a researcher-created measure to assess to what extent the behavioral task was completed and whether the client was able to act with self-compassion and awareness in response to carrying out (or not carrying out) the task. The treatment protocol is available upon request from the corresponding authors.

### 2.3 Measures

#### 2.3.1 Symptom distress

The Depression Anxiety Stress Scale-21 (DASS-21) is a short form of the original 42-item self-report measure (Lovibond and Lovibond, 1995). It is composed of three 7-item subscales which assess levels of depression, anxiety, and stress. The DASS-21 has been shown to be psychometrically sound across cultures (Bibi et al., 2020), possess adequate construct validity (Henry and Crawford, 2005), and demonstrate good to excellent reliability (Cronbach’s alphas of 0.88, 0.82, 0.90, and 0.93 for the Depression, Anxiety, Stress, and Total scales, respectively; Henry and Crawford, 2005).

#### 2.3.2 Psychological flexibility

The Acceptance and Action Questionnaire-II (AAQ-II) is a 7-item Likert-type scale which assesses psychological inflexibility and experiential avoidance (Bond et al., 2011). The measure has been found to be valid and reliable across race, age, and gender (Bond et al., 2011; Fledderus et al., 2012). Scores range from 7 to 49, with higher scores indicating higher psychological inflexibility. Bond et al. (2011) note that scores above 24–28 indicate high psychological inflexibility, which is associated with significant emotional distress.

#### 2.3.3 Self-compassion

The Self-Compassion Scale-Short Form (SCS-SF; Raes et al., 2011) is a 12-item Likert-type scale which assesses constructs related to self-compassion, including self-kindness, self-judgment, common humanity, isolation, mindfulness, and over-identification. It is recommended that the total scale score, ranging from 12 to 60, is used for research purposes since the subscale scores are less reliable in the short form. The scale has demonstrated good internal consistency (Cronbach’s alpha ≥0.86) and a high correlation with the long-form SCS (*r* = 0.97).

#### 2.3.4 Guilt, shame, detachment, and externalization of blame

The Test of Self-Conscious Affect (TOSCA-3; Tangney et al., 2000) is a self-report measure which requires individuals to rate how they would respond to hypothetical scenarios. While the full scale includes both negative and positive scenarios, Tangney et al. (2000) provide the option of implementing a short version of the scale by only including the negative scenarios. Participants in this study were administered the short version. Scores produce indices related to self-critical emotions: Shame-proneness, Guilt-proneness, Externalization of Blame, and Detachment. Detachment and Externalization of Blame are thought to represent cognitive processes that contribute to affective experiences of guilt and shame. At moderate levels, Detachment reflects a tendency to attribute failures to situational factors and/or a belief that mistakes and faults are acceptable. At higher levels, Detachment may reflect lack of concern.

#### 2.3.5 Quality of life

The 16-item Quality of Life Scale (Flanagan, 1978) is a Likert-type scale that assesses five conceptual domains of quality of life: material and physical well-being, relationships with other people, social, community and civic activities, personal development and fulfillment, and recreation. Scores range from 16 to 112 and the average total score for healthy populations (i.e., without psychiatric or medical diagnoses) is approximately 90 (Burckhardt and Anderson, 2003).

#### 2.3.6 Functioning in valued life contexts

**Figure 1:**
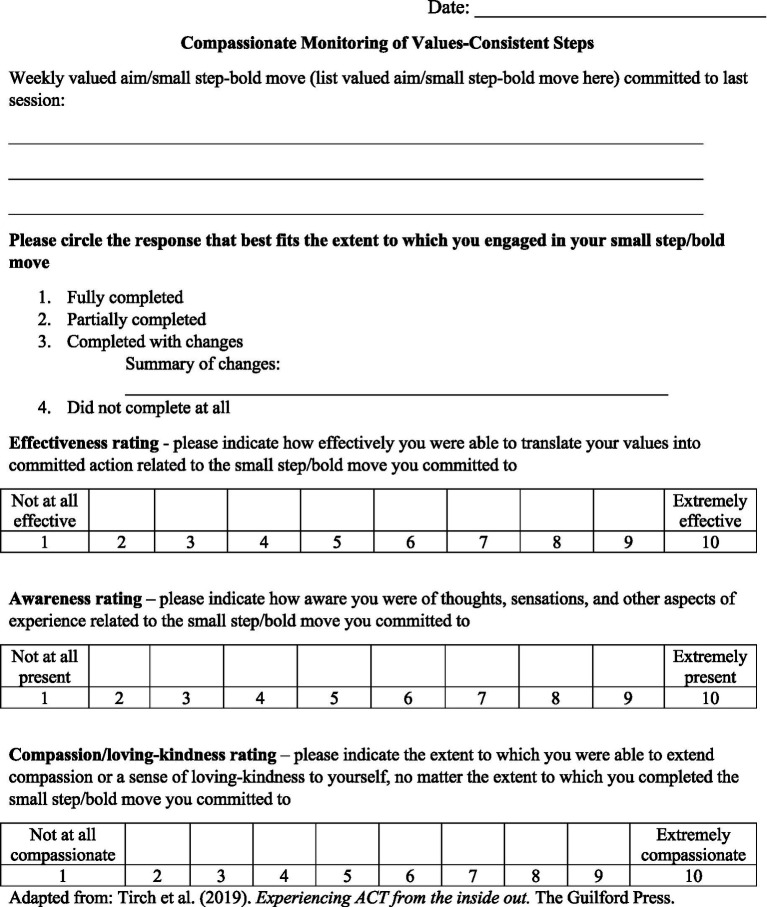
Functioning in valued life contexts measure.

A researcher-developed measure consisting of three Likert-type items was administered at the beginning of Sessions 4–16 to assess to what extent the participant-identified behavioral task was completed and to what extent the participant was able to act with self-compassion and mindful self-awareness in response to carrying out (or not carrying out) the task. See Figure 1 for measure items.

#### 2.3.7 Acceptability and receptivity toward the intervention

A researcher-developed questionnaire composed of four open-ended questions and six Likert-type items (scores ranging from 6 to 30) was administered to assess participants’ understanding of the primary principles of the intervention, which parts of the intervention participants found most and least helpful, whether participants had engaged in similar approaches prior to the study, participants’ perceived utility of the intervention, and participants’ impressions of the clinician.

#### 2.3.8 Clinician attitudes toward the intervention

The Perceived Characteristics of Intervention Scale (PCIS; Cook et al., 2015) is a measure composed of 20 Likert-type items that assesses clinicians’ perceptions of the intervention. The PCIS is interpreted by calculating two scores, one representing a single dimension of positive views toward the intervention (scores ranging from 18 to 90), and one representing perceived risk associated with the intervention (scores ranging from 2 to 10).

In addition, clinicians responded to an open-ended question assessing whether they have applied approaches or strategies consistent with CFACT in their own lives and how useful they perceive them to be.

### 2.4 Procedure

This study was approved by the University’s IRB. Treatment was administered by doctoral-level student clinicians through a telehealth video platform. Twenty-five percent of sessions within each case were chosen at random to be coded by an independent evaluator according to an adherence monitoring system developed based on recommendations by Plumb and Vilardaga (2010). Operationalization of therapeutic processes measured by the system was partly influenced by items in the ACT Fidelity Measure (ACT-FM; O’Neill et al., 2019). Cases that did not meet a predetermined criterion for sufficient adherence were excluded from analyses.

Following the brief phone screen, a 90-min intake session was conducted immediately prior to baseline for psychodiagnostic assessment and to confirm eligibility. Consistent with a multiple-baseline design, the initial point of implementation of CFACT varied based on the stability of that participant’s own baseline, during which participants did not receive any therapeutic intervention. Baseline phases spanned 3–6 weeks. Given the constraints of a community behavioral health clinic, baseline periods were capped at a maximum of 6 weeks.

Levels of psychological symptom distress (DASS-21), psychological flexibility (AAQ-II), and self-compassion (SCS-SF) were assessed weekly. As mentioned previously, a researcher-created assessment tool to track to what extent participants carried out their behavioral commitment task and how much self-compassion they experienced during the task was delivered weekly during the treatment phase. Levels of shame, guilt, detachment, externalization of blame (TOSCA-3), and perceived quality of life (QOLS) were assessed at the beginning of baseline and every 3–4 weeks during the treatment phase. Measures of perceived utility and acceptability were administered to both clients and clinicians at the end of the treatment phase. Participants were also asked to write brief narratives on their experience with the intervention at the end of the treatment phase to contextualize quantitative results and provide a deeper understanding of participants’ experiences.

### 2.5 Data analysis method

Data from the AAQ-II, DASS-21, and SCS-SF were examined through visual inspection (Kazdin, 2011; Kratochwill et al., 2010). Conservative dual-criterion (CDC; Fisher et al., 2003; Swoboda et al., 2010) analyses were also conducted to increase reliability of visual inspection. More specifically, two lines, one reflecting the linear trend in the baseline and one reflecting the mean of the baseline, were plotted. The lines were augmented by 0.25 standard deviations in the direction of the predicted treatment effect. If a minimum predetermined number of data points within the treatment phase (Fisher et al., 2003) were above or below both of the lines, depending on predicted treatment effect, it was concluded that a treatment effect had been demonstrated. In the case of missing data in the treatment phase, this predetermined number of data points was adjusted based on Fisher et al.’s (2003) criteria. Data from the TOSCA-3 and QOLS were also plotted and inspected visually for overall trajectories. Descriptive statistics of treatment adherence/integrity, receptivity, acceptability, perceived utility, and functioning in valued life contexts during treatment were analyzed.

Exploratory cross-lagged correlation analyses were conducted to determine if changes in psychological flexibility, self-compassion, and symptom distress were temporally associated. Symptom distress is typically conceptualized as an outcome variable, while psychological flexibility and self-compassion are often categorized as process variables. However, given that increased self-compassion and psychological flexibility may be conceptualized as desired outcomes in and of themselves in CFACT’s model of *compassionate flexibility*, we looked at temporal relationships between all three variables in both directions. The analyses were conducted with the Simulation Modeling Analysis software (Borckardt et al., 2008), which analyzes strength and temporality of associations, after adjusting for autocorrelation. We set analyses for +/− 5 lags, with 5,000 simulations, and a Bonferroni-corrected significance level of *p* < 0.05.

## 3 Results

### 3.1 Retention

Of 14 cases enrolled in the study, 11 (79%) participants completed the full protocol. As noted in Method, four cases were not included in the analyses due to baseline instability or technical difficulties related to telehealth and recording.

### 3.2 Treatment integrity

Recorded sessions from seven cases were evaluated for treatment integrity by an independent coder. Five cases (71%) met the established criterion for sufficient adherence to the treatment protocol, suggesting moderately high treatment integrity, in line with our hypothesis.

### 3.3 Clinician attitudes toward the intervention

Clinicians who implemented the full CFACT protocol with a participant completed the PCIS and related open-ended questions on acceptability and receptivity, regardless of whether their implementation of the protocol met treatment adherence criteria. Clinicians’ scores ranged from 63 to 73 (*M* = 68.50, *SD* = 4.43), suggesting moderately high ratings of the intervention, in line with the hypothesis. Ratings of items related to the following specific sub-constructs, with possible scores ranging from 2 to 10, were analyzed: relative advantage of the intervention compared to existing practices (*M* = 6.50, *SD* = 1.29), compatibility of the intervention with the clinician’s existing knowledge/values (*M* = 7.50, *SD* = 0.58), complexity (*M* = 7.75, *SD* = 0.50), trialability (*M* = 7.75, *SD* = 0.50), observability of results (*M* = 7.50, *SD* = 0.58), potential for reinvention/adaption of the intervention (*M* = 7.25, *SD* = 0.50), perceived utility (*M* = 7.50, *SD* = 1.00), whether the intervention can be effectively taught and learned (*M* = 8.25, *SD* = 1.26), and whether support components (manual, training materials) are helpful (*M* = 8.50, *SD* = 0.58). Ratings for perceived risk (*M* = 3.75, *SD* = 1.50) indicated clinicians believed risk associated with the intervention was low.

Clinicians’ responses to the open-ended question (assessing whether they have applied approaches/strategies consistent with CFACT in their own lives and how useful they perceive them to be) revealed that all found many of the messages, approaches, and exercises from CFACT helpful for their clients and clinicians often implemented these same approaches in their own lives. One half of clinicians reported they preferred implementing less structured therapies and/or experienced some difficulty adhering to the manualized nature of the CFACT protocol and the study-related procedures.

### 3.4 Acceptability and receptivity toward the intervention: Participant ratings and qualitative feedback

Results in this subsection are based on four participants’ results, since one participant did not submit the acceptability/receptivity questionnaire. Participant ratings on the Likert-type scale (*M* = 29.25, *SD* = 1.50) suggest very strong acceptability and receptivity toward CFACT, in line with hypotheses. Ratings of specific items (from 1 = *not at all* to 5 = *very much*) suggest all participants found the approach to be effective (*M* = 4.75, *SD* = 0.43), all were interested in continuing practicing elements of CFACT (*M* = 4.75, *SD* = 0.43), and all were “very likely” to recommend the treatment to others (*M* = 5.00, *SD* = 0.00). All participants reported that they did not think there was any risk of CFACT having a negative impact (*M* = 1.00, *SD* = 0.00). Finally, all participants provided high ratings for clinician knowledge (*M* = 4.75, *SD* = 0.43) and trustworthiness (*M* = 5.00, *SD* = 0.00).

In terms of qualitative feedback about CFACT, themes describing overall impressions of the therapy included: messages conveyed in CFACT were “transformative”; the therapy was effective in promoting increased self-compassion; the therapy was effective in modifying thinking and other ways of relating to the self; and CFACT was relevant and easily applied to daily life. Themes regarding what participants found most helpful about the treatment included: increasing self-compassion and reducing perfectionism and self-critical responses; in-session meditations and experiential practices; the non-judgmental, normalizing approach to all emotional experience; and the validating stance of the clinician. Themes relating to what participants found least helpful included: the structured nature of the protocol (one participant stated she was looking for a less structured, general supportive/talk therapy) and completing weekly measures as part of the study procedure. One participant stated that “nothing” was unhelpful. Three-fourths of the participants reported having some experience with related approaches prior to engaging in CFACT, including mindfulness or meditation-related practice. One participant stated she had previously practiced mindfulness, but she had not found mindfulness to be helpful until practicing it in the context of CFACT.

### 3.5 Symptom distress

**Figure 2:**
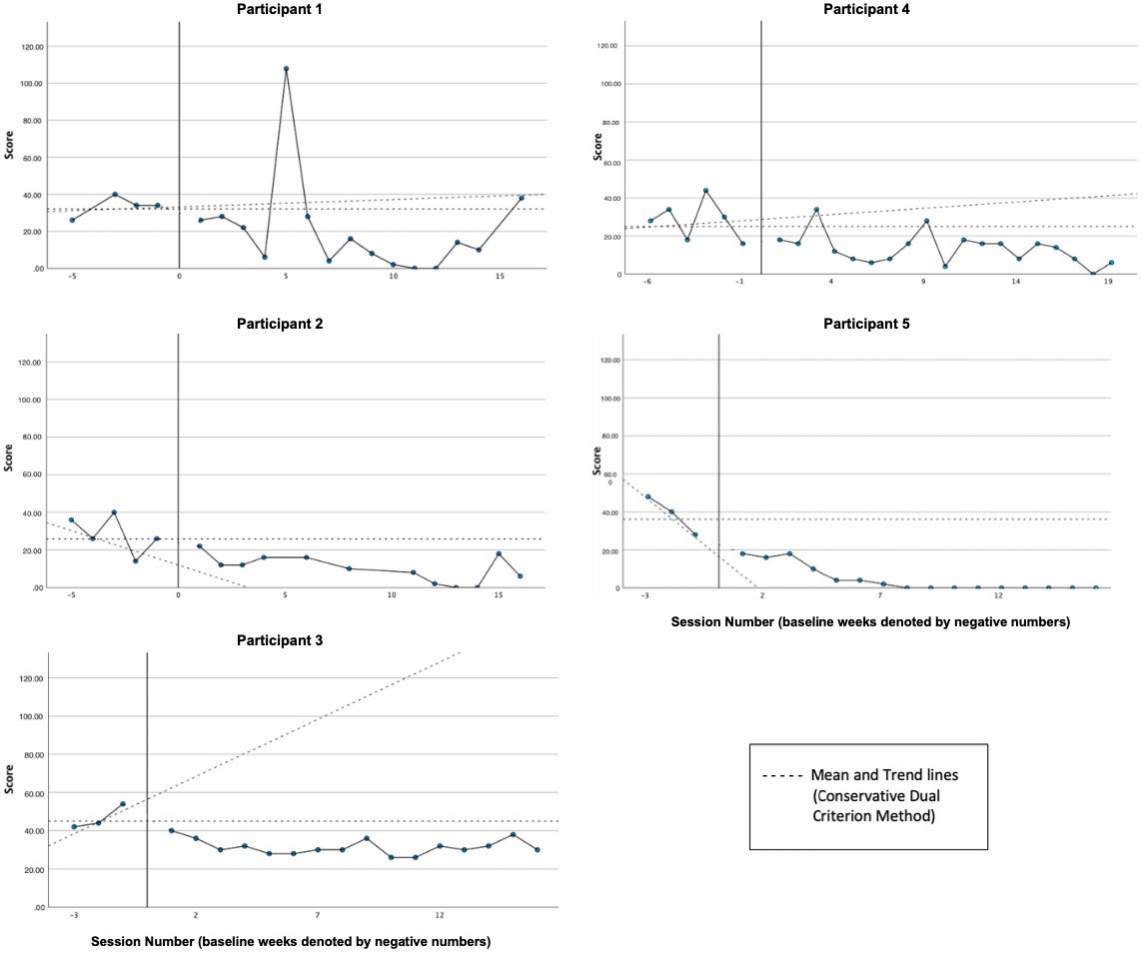
Symptom distress (DASS-21).

Graphs of symptom distress across participants, as measured by the DASS-21, are presented in Figure 2. Based on visual and CDC analyses, reliable decreases in distress were exhibited by Participant 1 (P1), Participant 3 (P3), and Participant 4 (P4). While both P2’s and P5’s treatment phase ratings (P2 [*M* = 10.16, *SD* = 7.20]; P5 [*M* = 4.50, *SD* = 6.91]) are all clearly below the baseline mean (P2 [*M* = 28.4, *SD* = 10.14]; P5 [*M* = 38.66, *SD* = 10.06]), neither could be analyzed reliably by the CDC method since baseline data were trending in the direction of expected treatment effect, creating a floor effect. Overall, in line with our hypothesis, all participants exhibited decreases in average distress levels and, according to CDC analyses, reliable treatment effects were demonstrated for all three cases (P1, P3, and P5) whose baselines were sufficiently stable.

### 3.6 Psychological flexibility

**Figure 3:**
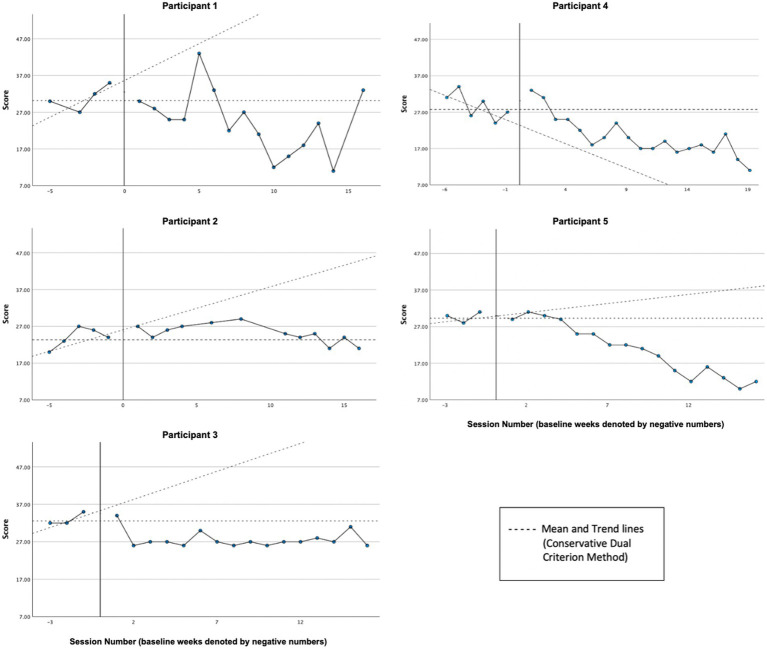
Psychological in/flexibility (AAQ-II).

Graphs of psychological inflexibility across participants, as measured by the AAQ-II, can be found in Figure 3. A reliable treatment effect was demonstrated by P1, P3, and P5 based on visual analysis and the CDC method. Visual inspection by the mean demonstrates P4’s inflexibility decreased from baseline (*M* = 28.67, *SD* = 7.09) to treatment phase (*M* = 20.21, *SD* = 5.50). However, P4’s data cannot be analyzed reliably by the CDC method since baseline data were trending in the direction of expected treatment effect, creating a floor effect. A reliable treatment effect was not demonstrated for P2, whose level of psychological in/flexibility remained fairly stable and at the bottom end of the threshold for “high psychological inflexibility” across baseline (*M* = 24.00, *SD* = 2.74) and treatment phases (*M* = 25.08, *SD* = 2.50). Overall, four of five cases demonstrated decreases in average psychological inflexibility, in line with our hypothesis. Of the four cases whose baselines demonstrated sufficient stability for CDC analysis (P1, P2, P3, P5), three (P1, P3, P5) exhibited a reliable treatment effect.

### 3.7 Self-compassion

**Figure 4:**
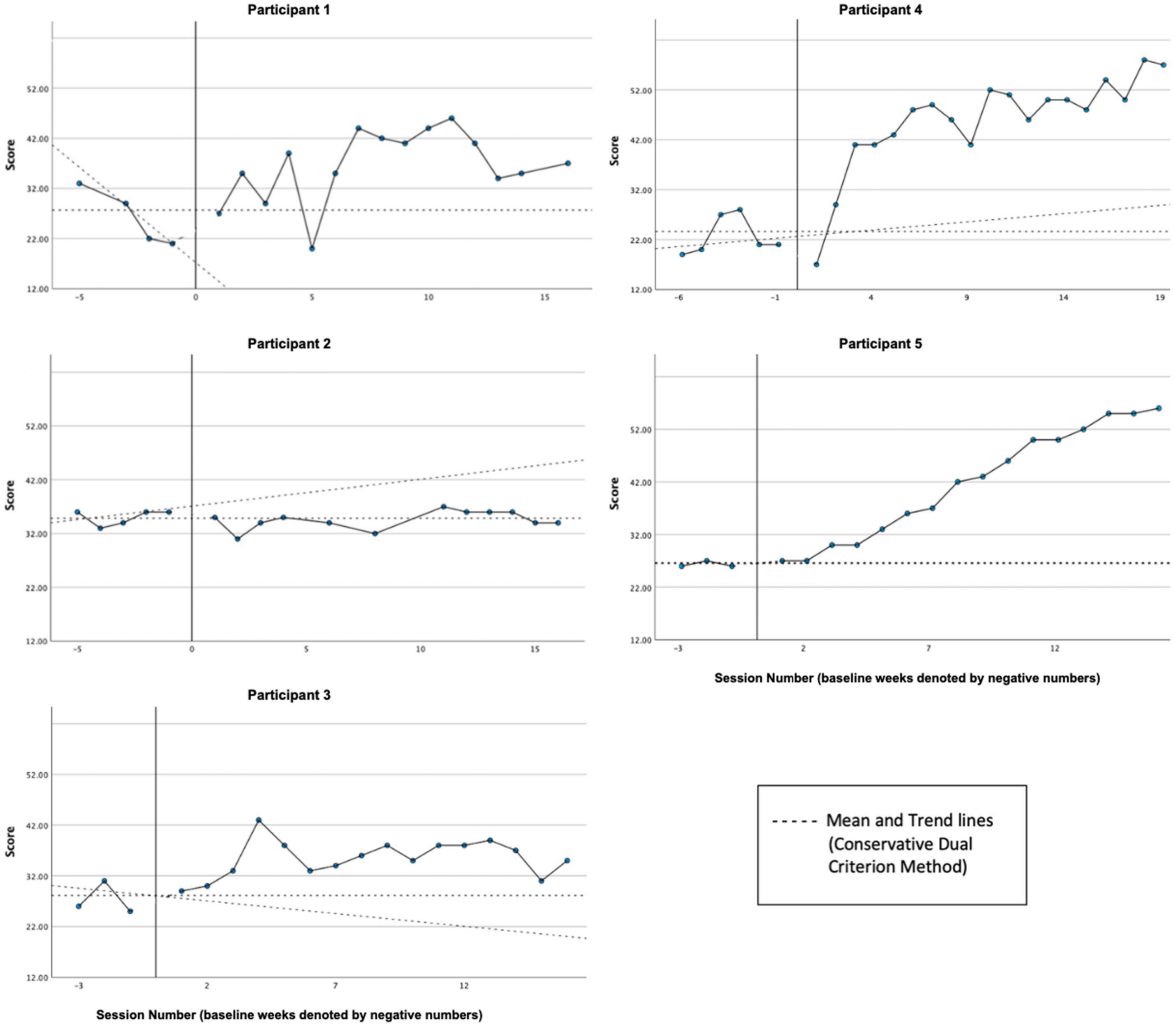
Self-compassion (SCS-SF).

Graphs of self-compassion, as measured by the SCS-SF, across participants are presented in Figure 4. In line with the hypothesis, visual and CDC analyses indicated reliable treatment effects for the majority of participants (P1, P3, P4, and P5), with overall self-reported self-compassion increasing from baseline to treatment phase. P2’s self-reported self-compassion did not appear to change reliably from baseline (*M* = 35.00, *SD* = 1.41) to treatment phase (*M* = 34.5, *SD* = 1.73).

### 3.8 Temporal associations between psychological flexibility, self-compassion, and distress

The results of the supplemental cross-lagged correlation analyses can be found in Table 2. Overall, results did not indicate consistent predictive associations between the variables. Psychological flexibility and self-compassion were highly correlated at Lag 0 (concurrent change) across most participants. Results by participant are reported in section 3.12.

**Table 2:** Cross-lagged correlations.

		**Lag 0**		**Lag + 1**		**Lag -1**		**Lag -2**	
**Participant**	**Correlation**	***r***	***p***	***r***	***p***	***r***	***p***	***r***	***p***
1	Flexibility ➔ distress	.82	<.001						
	Self-compassion ➔ distress	-.76	.001						
	Flexibility ➔ self-compassion	-.72	.002						
2	Flexibility ➔ distress								
	Self-compassion ➔ distress								
	Flexibility ➔ self-compassion								
3	Flexibility ➔ distress	.79	<.001						
	Self-compassion ➔ distress	-.68	.002						
	Flexibility ➔ self-compassion	-.69	.003						
4	Flexibility ➔ distress								
	Self-compassion ➔ distress								
	Flexibility ➔ self-compassion	-.91	<.001			-.78	.001		
5	Flexibility ➔ distress	.81	.003					.74	.004
	Self-compassion ➔ distress	-.82	.004			-.79	.003		
	Flexibility ➔ self-compassion	-.98	<.001	-.85	.002	-.85	.001		

Positive lag indicates a change in the first variable listed in a pair predicted change in the second variable. Negative lag indicates the second variable in a pair predicted change in the first. Zero lag indicates a contemporaneous correlation.

### 3.9 Guilt, shame, detachment, and externalization of blame

#### 3.9.1 Guilt

**Figure 5:**
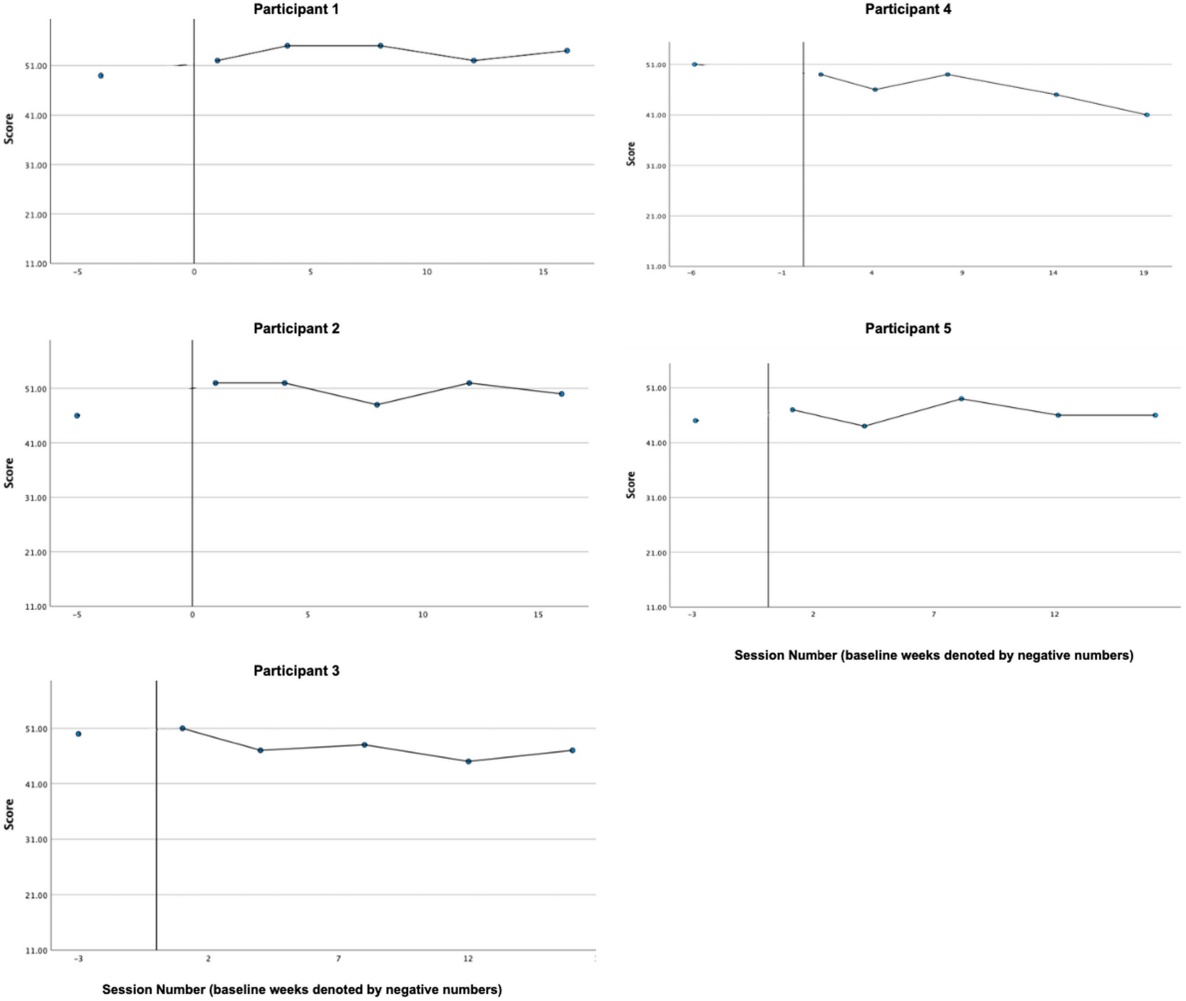
Guilt-proneness (TOSCA-3 short).

Graphs of guilt-proneness, as measured by the TOSCA-3, across participants are presented in Figure 5. Contrary to the hypothesis, the majority of participants’ ratings of guilt-proneness during the treatment phase did not appear to decrease significantly relative to baseline. Treatment phase guilt ratings for P1 (*M* = 53.60, *SD* = 1.52) and P2 (*M* = 50.80, *SD* = 1.79) were higher compared to pretreatment ratings (P1: 49.00, P2: 46.00), while P3’s (*M* = 47.60, *SD* = 2.19) and P4’s (*M* = 46.00, *SD* = 3.32) treatment phase ratings were lower compared to the pre-treatment score (P3: 50.00, P4: 51). These changes were all slight, however. P5’s treatment phase ratings (*M* = 46.4, *SD* = 1.82) were similar to their pretreatment rating (45). Importantly, these results, as well as those of the other TOSCA-3 subscales and of the QOLS, should be interpreted with caution since analyses are based on a single pretreatment rating, rather than a full, stable baseline.

#### 3.9.2 Shame

**Figure 6:**
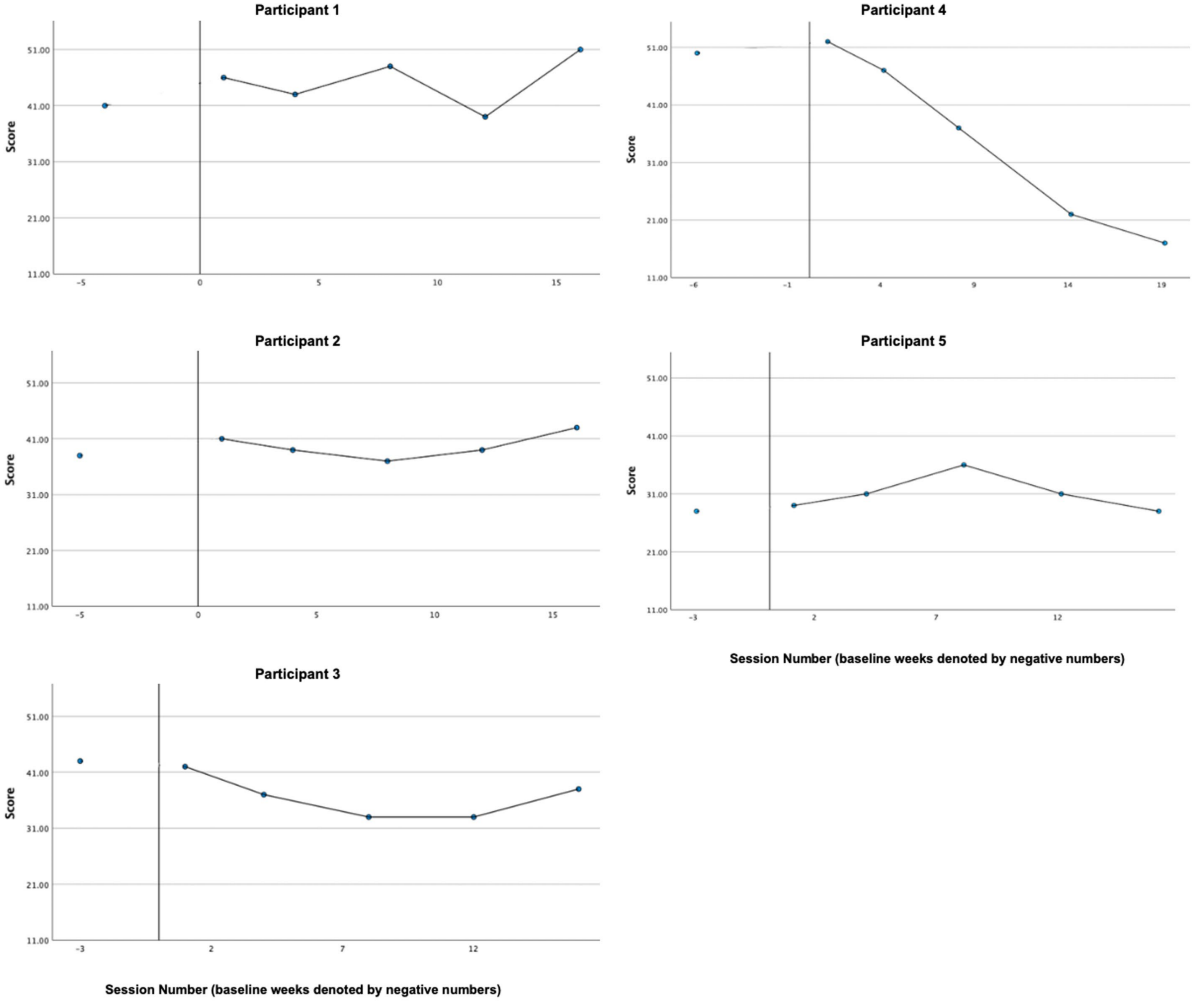
Shame-proneness (TOSCA-3 short).

Graphs of shame-proneness, as measured by the TOSCA-3, across participants are presented in Figure 6. Data trajectories were mixed. Both P3’s and P4’s treatment phase shame ratings (P3 [*M* = 36.60, *SD* = 3.78], P4 [*M* = 35.00, *SD* = 15.25]) were lower compared to pre-treatment (P3: 43.00, P4: 50), in line with our hypothesis. P4’s decrease in shame-proneness is especially pronounced. However, treatment phase shame ratings for P2 (*M* = 39.80, *SD* = 2.28) and P5 (*M* = 31, *SD* = 3.08) were similar to pre-treatment (P2: 38.00, P5: 28), and P1’s (*M* = 45.40, *SD* = 4.62) was slightly higher compared to pre-treatment (41.00).

#### 3.9.3 Detachment

**Figure 7:**
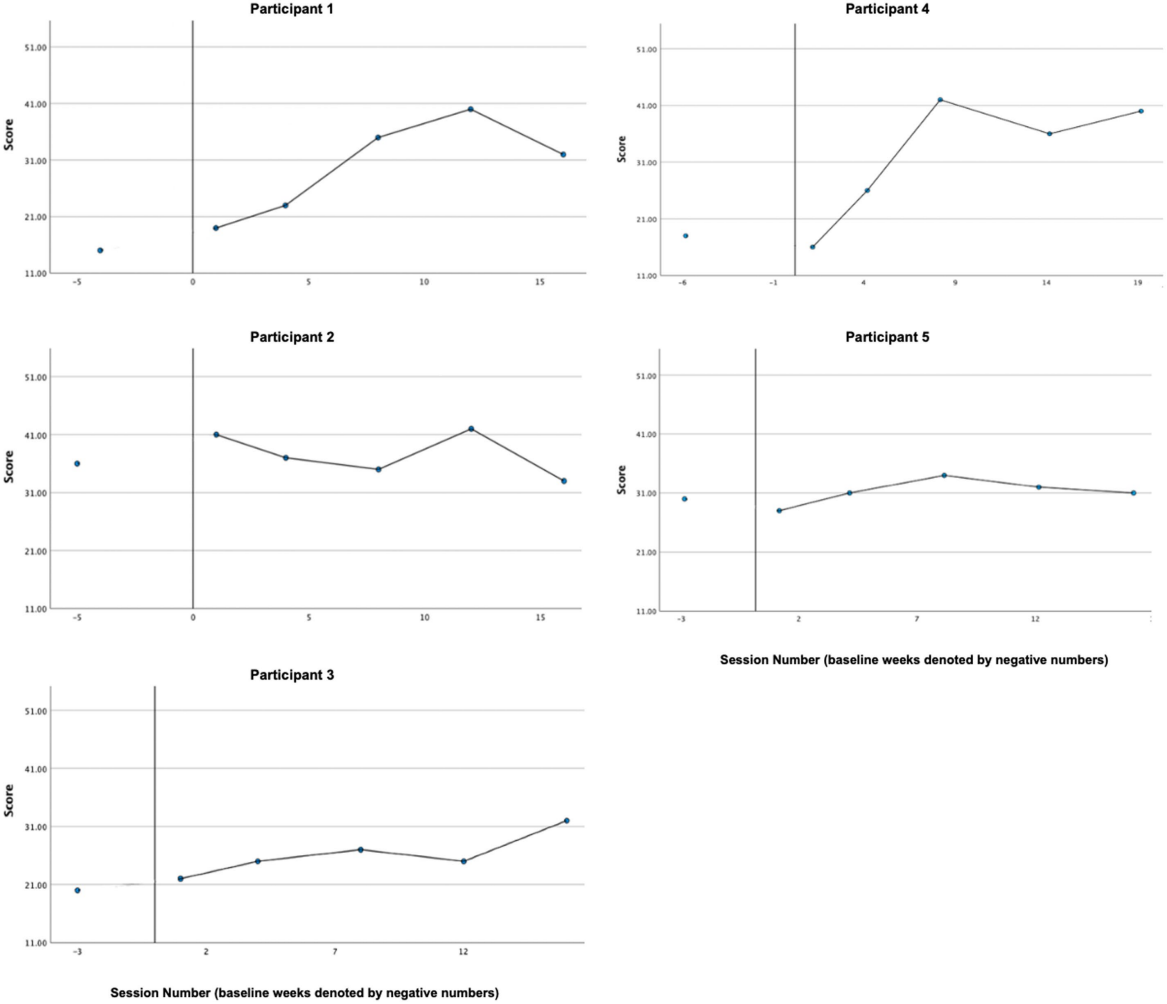
Detached (TOSCA-3 short).

Graphs of detachment, as measured by the TOSCA-3, across participants are presented in Figure 7. In line with our hypothesis, an increase in detachment was observed in the majority of participants (P1, P3, and P4) from pre-treatment to treatment phase. P2’s and P5’s detachment scores were similar across pre-treatment and treatment phases.

#### 3.9.4 Externalization of blame

**Figure 8:**
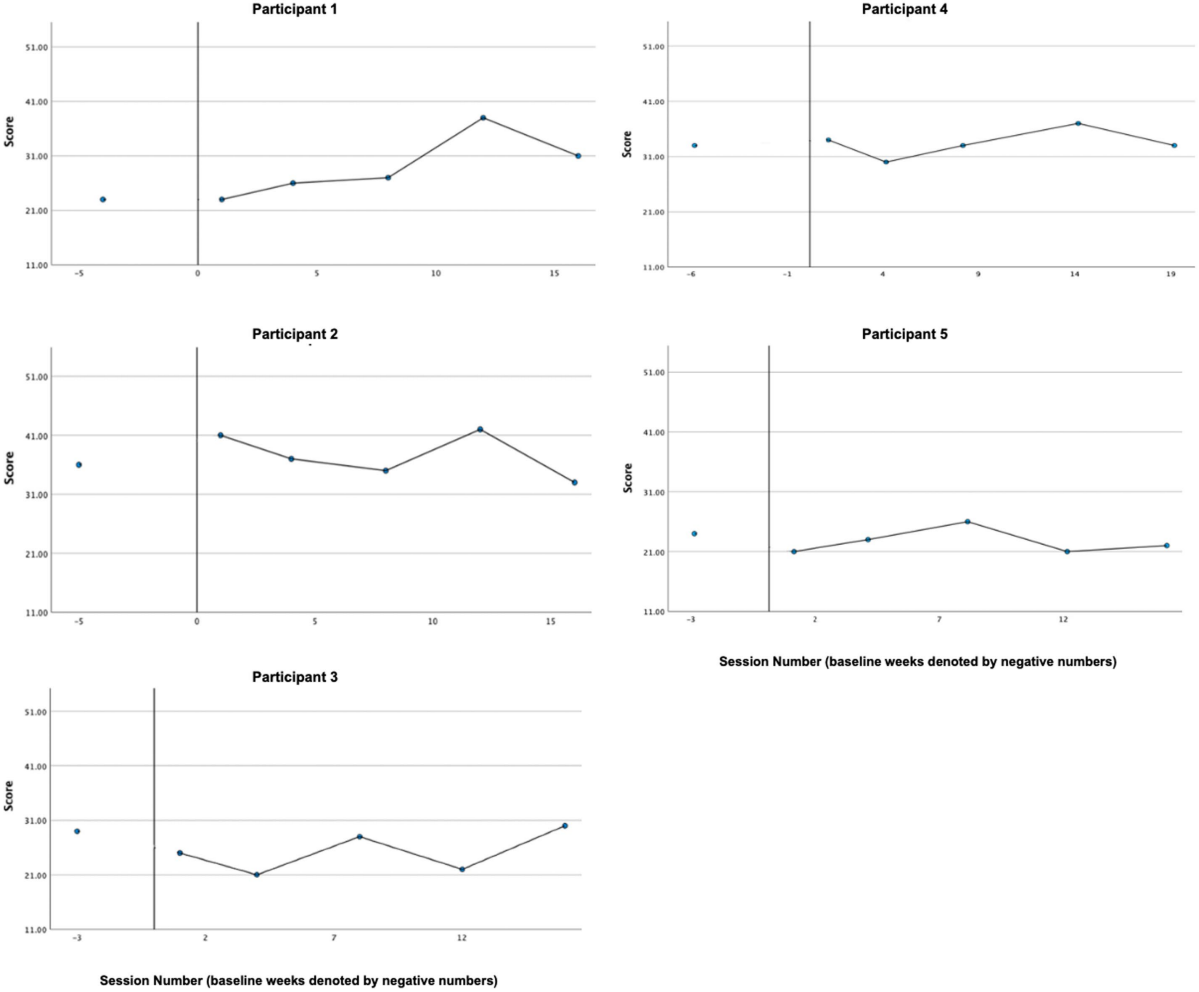
Externalization of blame (TOSCA-3 short).

Graphs of externalization of blame, as measured by the TOSCA-3, across participants are presented in Figure 8. Contrary to hypotheses, participants did not exhibit clear decreases in externalization. All participants’ ratings were low to moderate at pretreatment and remained in this range during the treatment phase.

### 3.10 Quality of life

**Figure 9:**
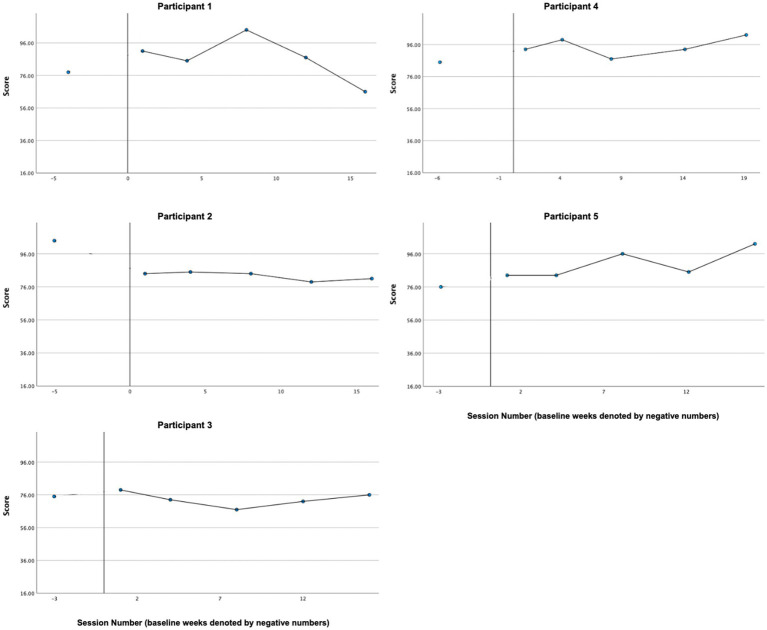
Quality of life (QOLS).

Graphs of quality of life, as measured by the QOLS, across participants are presented in Figure 9. Results were mixed. Both P4 and P5’s quality of life ratings increased from pre-treatment (P4: 85, P5: 76) to treatment phase (P4 [*M* = 94.8, *SD* = 5.85], P5 [*M* = 89.8, *SD* = 8.70]). While P1’s average treatment phase rating (*M* = 86.60, *SD* = 13.69) was also higher than at pretreatment (78.00), the treatment phase data were highly variable, with a clear upward trend in the beginning of treatment, switching to a downward trend during the second half of treatment. Treatment phase ratings for P3 (*M* = 73.40, *SD* = 4.51) were similar to ratings at pretreatment (P3: 75.00,). P2’s quality of life rating decreased between pretreatment (104.00) and the initiation of treatment and remained relatively stable throughout treatment (*M* = 82.60, *SD* = 2.51).

### 3.11 Functioning in valued life contexts

**Figure 10:**
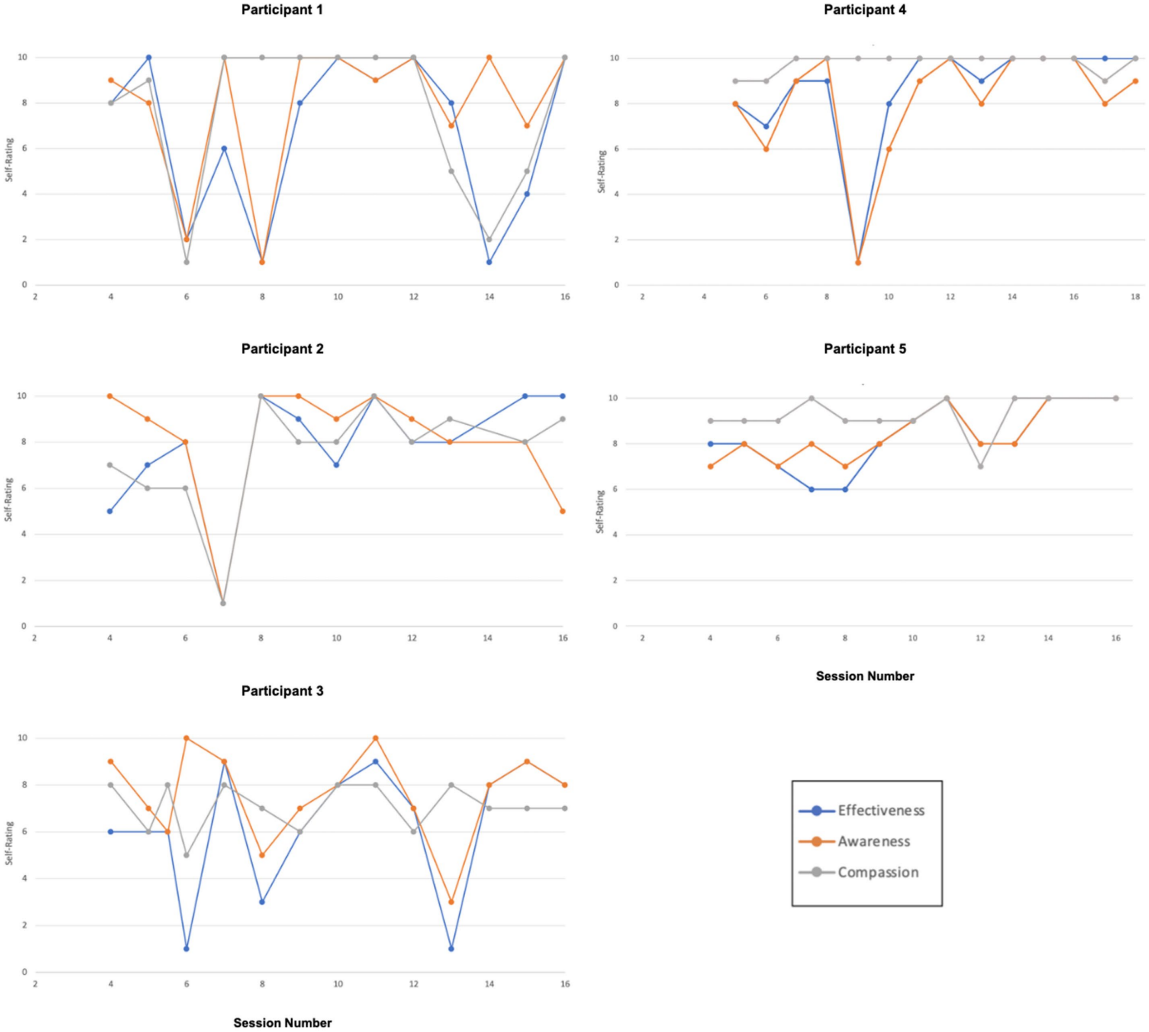
Functioning in valued life contexts.

Participants provided ratings in Session 4–16 of effectiveness, awareness, and self-compassion related to a values-consistent behavioral task set in the previous session. Full descriptive statistics can be found in Table 3. The percentage of behavioral goals reported “fully completed,” “partially completed,” or “completed with changes” ranged from 80 to 100% indicating a high degree of engagement in between-session value-aligned behaviors. The percentage of behavioral goals reported as “not completed” ranged from 0 to 20%. Average self-rated effectiveness scores ranged from 6.21 to 8.64 out of a maximum of 10, indicating moderately high effectiveness. Average self-rated awareness scores ranged from 7.57 to 8.33, indicating moderately high awareness. Average self-rated self-compassion scores ranged from 7.07 to 9.79, indicating moderately high to high self-compassion. Graphs of effectiveness, awareness, and self-compassion ratings are presented in Figure 10 and demonstrate that trajectories in these three scales over time varied by participant. Intrapersonal relationships between self-rated effectiveness, awareness, and self-compassion also varied by participant.

**Table 3:** Behavioral goal (‘Small Step’) ratings.

**Participant**	**P1**	**P2**	**P3**	**P4**	**P5**
Percentage reported ‘fully completed’, ‘partially completed,’ or ‘completed with changes’	92.3%	80.0%	85.7%	92.9%	100%
Percentage reported as ‘not completed’	7.7%	20.0%	14.3%	7.1%	0%
Average effectiveness	6.69 (*SD*=3.52)	7.75 (*SD*=2.63)	6.21 (*SD*=2.75)	8.64 (*SD*=2.41)	8.17 (*SD*=1.40)
Average awareness	7.92 (*SD*=3.07)	8.08 (*SD*=2.64)	7.57 (*SD*=1.95)	8.14 (*SD*=2.48)	8.33 (*SD*=1.15)
Average self-compassion	7.69 (*SD*=3.30)	7.50 (*SD*=2.43)	7.07 (*SD*=1.00)	9.79 (*SD*=0.43)	9.25 (*SD*=0.87)

### 3.12 Results by participant

#### 3.12.1 Participant 1

Overall reliable improvements in symptom distress, self-compassion, and psychological flexibility for P1 were demonstrated based on visual analysis and the CDC method. While all three variables were correlated at Lag 0, there was no evidence of predictive association. Notably, data demonstrated deterioration after Session 12, correlating with a significant increase in reported life stressors toward the end of treatment. Ratings of shame-proneness and quality of life mirrored these trajectories, exhibiting an initial improvement, followed by a deterioration toward the end of treatment. Detachment increased, as hypothesized. P1 also exhibited slight overall increases in guilt and externalization. Engagement in value-consistent behavioral actions was high. Despite these inconsistent patterns over the course of the treatment phase, P1 provided high ratings of acceptability and receptivity.

#### 3.12.2 Participant 2

Improvements across symptom distress, psychological flexibility, self-compassion, quality of life, or the TOSCA-3 scales were not observed. Yet, P2 provided maximum ratings for acceptability and receptivity and reported that engaging in CFACT resulted in significant changes in perspective. Cross-lagged correlational analyses yielded no significant associations between psychological flexibility, self-compassion, and symptom distress.

#### 3.12.3 Participant 3

P3’s data indicated reliable treatment effects in symptom distress, psychological flexibility, and self-compassion. While all three variables were correlated at Lag 0, there was no evidence of predictive association. Also, in line with hypotheses, shame appeared to decrease and detachment increased. No significant effects were observed in quality of life, guilt, or externalization of blame. P3 reported maximum acceptability and receptivity scores.

#### 3.12.4 Participant 4

Overall, analyses indicated reliable treatment effects in symptom distress and self-compassion for P4. While a consistent decrease in psychological inflexibility across treatment was apparent, a floor effect created by the baseline trend precluded conducting CDC analysis to determine if the change was reliable. Neither psychological flexibility nor self-compassion was correlated with symptom distress. However, psychological flexibility and self-compassion were strongly correlated at Lag 0, indicating a contemporaneous relationship, and at Lag −1, though this may be an artifact of a strong correlation at Lag 0. Visual inspection of data suggests a large decrease in shame, a large increase in detachment, a slight decrease in guilt-proneness, and a slight increase in quality of life, all in line with predicted treatment effects. Engagement in value-consistent behavioral actions was high overall, with related self-compassion remaining consistently high regardless of fluctuations in perceived effectiveness and awareness. A clear change was not observed in externalization of blame. P4 reported maximum acceptability and receptivity scores.

#### 3.12.5 Participant 5

P5’s data indicated reliable treatment effects for psychological flexibility and self-compassion. Though a consistent decrease in symptom distress was observed across treatment, a floor effect was created by the baseline trend, rendering the treatment effect unreliable based on the CDC analysis method. Psychological flexibility, self-compassion, and symptom distress all strongly correlated with each other at Lag 0. Flexibility and self-compassion also correlated with distress at Lag −1 and Lag −2, indicating that changes in symptom distress predicted changes in the other two variables. Analyses also yielded significant correlations between psychological flexibility and self-compassion at Lags +1 and −1, though this may be an artifact of a nearly perfect correlation at Lag 0. A clear increase in quality of life was reported. Clear treatment effects were not observed in guilt, shame, detachment, or externalization of blame. Engagement in value-consistent behavioral actions was high overall.

### 3.13 Relationships between pre-intervention self-compassion and shame-proneness, and outcomes

P1, P3, P4, and P5 all reported low self-compassion scores at pre-treatment, while P2’s was relatively higher. As predicted, those with lower self-compassion at pre-treatment appeared to benefit more from CFACT, as evidenced by a higher number of reliable treatment effects observed across measured variables. The relationship between pre-treatment shame-proneness and response to CFACT was not as consistent across participants. These associations should be interpreted with caution, however, since differences in shame and self-criticism at pre-treatment between participants were slight.

## 4 Discussion

A non-concurrent multiple baseline design was implemented at a training clinic to examine the acceptability, receptivity, and preliminary outcomes of a 16–19-session individual therapy protocol of CFACT developed to address psychological difficulties transdiagnostically. Client trajectories of reported symptom distress, self-compassion, guilt, shame, detachment, externalization of blame, psychological flexibility, functioning in valued life contexts, and quality of life were analyzed. Cross-lagged correlation analyses were conducted to examine temporal associations between changes in self-compassion, psychological flexibility, and distress. Additionally, ratings of and qualitative feedback on acceptability and receptivity were collected from both participants and clinicians.

Treatment integrity was moderately high. Overall, clinicians provided moderately high ratings of the intervention. Clinicians agreed that the intervention was understandable, aligned with their clinical judgment and how they like to work, improved the quality of the clinicians’ work, could be effectively taught and learned, and that the manual and training materials were helpful. They also agreed that improvements in patients were observable. On average, clinicians provided moderate ratings regarding whether CFACT was more effective or convenient compared to other treatments they have implemented. Finally, clinicians believed the intervention posed moderately low risk to patients.

Results suggest participant acceptability and receptivity toward the intervention were high, with all respondents saying they were very likely to recommend the treatment to others. When asked about their impressions of the therapy, participants reported CFACT was effective in promoting self-compassion and modifying how they related to themselves and to others. Participants stated several elements of the therapy, including the focus on increasing self-compassion, in-session mindfulness practices, and the validating stance of the therapist, were most helpful. Most respondents engaged in related practices prior to the intervention, with one participant stating that these practices had not been helpful until practiced in the context of CFACT. Further, the 21% total dropout rate (79% retention) also suggests high participant acceptability. Notably, this dropout rate is lower than the average for CBT treatment evaluation studies (15.9% dropout at pretreatment and 26.2% during treatment; Fernandez et al., 2015).

Fifty percent of clinicians and one participant stated they believed the protocol was too structured. As a process-based therapy, CFACT is meant to be implemented flexibly. Nevertheless, this may have been limited by the manualized nature of the protocol. We believe that shifting to a more modular protocol format, which would allow the clinician to extend/shorten time spent on certain interventions based on the individual patient’s needs, would promote a balance of adherence to the treatment and flexibility in implementation. This would likely increase acceptability and receptivity for both patients and clinicians and may, in turn, improve effectiveness of the treatment.

Impacts of the intervention, as measured by quantitative self-report instruments, were mixed. In line with hypotheses, all participants exhibited decreases in average distress, with reliable decreases observed in all three cases whose baselines were sufficiently stable for CDC analysis. Four of five cases demonstrated decreases in average psychological inflexibility. Moreover, three of the four cases whose baselines demonstrated sufficient stability for CDC analysis exhibited reliable decreases in psychological inflexibility. Four of five participants exhibited reliable increases in self-compassion.

Results indicated improvements in psychological flexibility were associated with concurrent improvements in self-compassion across most participants. For three of five participants, improvements in both psychological flexibility and self-compassion were associated with concurrent decreases in symptom distress. Overall, there was no clear evidence of predictive associations between the variables.

Guilt-proneness remained largely unchanged across pre-treatment and treatment phases, contrary to our hypothesis. It should be noted that the TOSCA-3 Guilt-Proneness scale measures mild or adaptive forms of guilt and has been shown to correlate positively with empathy (Luyten et al., 2002), which is promoted in CFACT as an attribute of compassion. It is possible that this lack of apparent change in guilt, and even slight increases in some participants, reflects increased empathy and motivation for reparative action, which align with the two dimensions of compassion promoted in CFACT (i.e., compassionate attention to suffering and motivation to prevent or alleviate suffering; Gilbert and Procter, 2006; Tirch et al., 2014).

Trajectories of shame-proneness were also mixed. Decreases in shame-proneness are purported to be a primary therapeutic process underlying CFT and, relatedly, CFACT (Braehler et al., 2013; Gilbert, 2009a). In line with this, trajectories of most participants’ shame-proneness generally mirrored treatment effects across other variables. While P5’s lack of change in shame ratings is seemingly incompatible with the improvements seen in most other variables, this may be explained by a low shame-proneness score at baseline creating a floor effect.

In line with hypotheses, detachment increased in most participants (P1, P3, and P4). Given research indicating higher detachment correlates with lower shame and with higher resilience (Luyten et al., 2002; Uji et al., 2011), these increases suggest a positive therapeutic impact. Two participants (P2 and P5) did not exhibit increased detachment. Notably, their pretreatment ratings were significantly higher than those of other participants. It is possible P2 and P5 already exhibited an adaptive level of detachment at pre-treatment, resulting in a ceiling effect.

Contrary to our hypothesis, participants did not exhibit clear decreases in externalization of blame. This should be interpreted with caution, though, since pre-treatment ratings were in the low to moderate range and remained relatively low across treatment phases.

Results for quality of life varied. Notably, this is despite moderately high engagement in values-consistent behavioral tasks across participants during treatment. It is possible that the addition of one weekly behavioral task was not sufficient to impact reported quality of life in all participants. Alternatively, participants may have already been engaging in value-consistent actions prior to treatment, so the increase in behavioral engagement during treatment may not have been robust enough to render a significant change in reported life quality.

Self-rated effectiveness, awareness, and self-compassion connected to the participant-identified values-aligned behavioral task were all moderately high to high. Patterns of these three ratings over the course of treatment varied by participant. Additionally, relationships between these variables within participants varied, with high levels of coupling/correlation in some participants’ data, and significant decoupling (independence) in other participants’ data. Further examination of these variables, as well as the other process and outcome variables mentioned above, in larger samples is needed to determine if there are broader patterns of relationships (i.e., mediators and moderators).

Due to the small sample size and the study design used, it is not possible to make generalizations or statistical inferences regarding whether certain participant characteristics correlated with treatment effect. However, relationships can be analyzed descriptively. Participant characteristics did not appear to be related to acceptability and receptivity, which were high across all participants. Treatment effect, as measured by quantitative outcome and process measures, may be related to pre-treatment levels of psychological inflexibility, and self-criticism/self-compassion, consistent with Leaviss and Uttley’s (2015) finding that those exhibiting higher self-criticism benefit most from compassion-focused interventions Notably, the only participant who did not exhibit improvements across most variables (P2) started with high levels of self-compassion and a level of psychological inflexibility below the threshold for clinically significant distress (Bond et al., 2011). Pre-treatment shame proneness, which has been demonstrated to be correlated with self-compassion/self-criticism and has also been shown to be a predictor of response to compassion-focused interventions (Leaviss and Uttley, 2015), was not as consistently connected to treatment effect in this study. In future studies, researchers may consider conducting moderation analyses or include larger samples of participants exhibiting wider ranges of shame and self-criticism at pre-treatment to evaluate this more reliably.

### 4.1 Limitations and considerations for future directions

This study was subject to several limitations. Participants did not receive any compensation for completing weekly measures, which may have impacted motivation and perceived burden. Relatedly, due to reliance on repeated self-report measures, data were subject to reporter bias. To mitigate these limitations in future studies, multimethod and multisource evaluation may be used to enhance the validity of assessment (Smith, 2012). Additionally, less frequent administration of measures, which has been implemented by some researchers (e.g., Petrocchi et al., 2021), could decrease participant burden, though internal validity may, in turn, be reduced in a single case experimental design (Chambless and Hollon, 1998; Kazdin, 2011).

We were unable to reliably analyze data in several instances where baselines did not exhibit sufficient stability, or where baseline ratings created substantial floor effects. Relatedly, it was not possible to establish stable data baselines for the TOSCA-3 and QOLS since they were only administered once at pretreatment. Therefore, inferences regarding treatment impact on shame, guilt, detachment, externalization of blame, and quality of life should be considered with caution. Calculation of reliable change indices (Jacobson and Truax, 1991) in future studies may allow for evaluation of outcomes in single case designs, even if a stable baseline is not established.

Single-case experimental design is recommended for examination of new treatments (Hayes et al., 2013). Despite this, methods for evaluating data, including general visual inspection and the CDC method, may not be ideal for examining multi-session treatment packages, such as CFACT. The CDC method and some criteria for visual inspection, such as latency of change and degree of overlap, assume that impacts of an intervention, if present, should be exhibited soon after the introduction of the intervention. However, in CFACT, treatment components are introduced sequentially over a 16-session protocol. It is possible that a sufficient dose of CFACT is not delivered immediately, but may take several sessions to cumulatively impact targeted processes. It is, therefore, possible that evaluating data strictly according to visual inspection criteria or according to the more systematic CDC method could obscure true treatment effects. Furthermore, the CDC method is especially stringent. While this reduces the chance of Type I error, it may underestimate true effects. Analyses of overall trajectories, as well as qualitative and observational data, may provide a more comprehensive and accurate understanding of a novel treatment during preliminary investigation.

Single case experimental design allows for participants to serve as their own controls. However, without an active placebo during baseline, it is not possible to discriminate between the impacts of CFACT and those due to non-specific factors such as clinician contact.

Certain participant characteristics may have impacted results as well. One participant did not meet criteria for a clinical disorder (instead receiving a *DSM-5* V-code) and some participants provided subclinical ratings of different process and/or outcome variables during baseline. It is possible that patients exhibiting higher distress would benefit more from CFACT or their treatment effects would be more discernible. Future studies should examine the impact of CFACT on individuals of a wider range of distress levels, as well as include participants of greater demographic diversity, to fully understand how these variables interact with treatment effect.

Relatedly, it is important to note that three-fourths of participants reported having some experience with related approaches, though we do not know much about the nature or extent of this exposure. Given that mindfulness is commonly discussed in the popular press (Stofleth and Manusov, 2019) and digital applications and programs that aim to foster mindfulness and self-compassion are widely used (Bégin et al., 2022), it is not surprising that most participants reported some exposure to these ideas or practices. Future research should aim to more precisely assess parameters of this exposure, and group designs might examine these as moderators of response to interventions.

This study only presented participant data collected through the treatment phase. Studies of future iterations of CFACT should also include an assessment at follow-up in order to elucidate whether and which impacts of CFACT persist over a longer-term period.

## 5 Conclusion

This study is the first to contribute to the evidence base for CFACT as a transdiagnostic treatment for anxiety, depression, and trauma-related difficulties. Overall, the results provide strong support for acceptability and receptivity toward CFACT across both participants and clinicians. Further, the study demonstrates that training clinicians in CFACT and implementing the treatment over telehealth in a training clinic setting was feasible. While quantitative results, as measured by outcome and process measures, were mixed, this study provides preliminary evidence that CFACT is effective in reducing symptom distress and increasing psychological flexibility, self-compassion, and detached attributional style. However, several limitations should be considered when interpreting these findings. Preliminary results suggest CFACT is a promising intervention, though additional research is warranted to gain a better understanding of its potential therapeutic impacts.

## Data Availability

The raw data supporting the conclusions of this article will be made available by the authors, without undue reservation.
